# Antecedents of Deviant Behavior: Psychological and Non-Psychological Factors and Ethical Justifications

**DOI:** 10.1007/s10672-021-09387-x

**Published:** 2021-09-11

**Authors:** Emiliano Di Carlo

**Affiliations:** University of Rome ‘Tor Vergata’, Via Columbia 2, 00133 Rome, Italy

**Keywords:** Common Good, Deviant Behavior, Ethical Reasoning, Ethical Theories, Moral Rationalization, Real Entity Theory, Virtue Ethics

## Abstract

An integrative model of ethical justifications in organizations is proposed. The model recognizes the roles of psychological and non-psychological factors on the link between ethical theories and the ethical reasoning and, consequently, on the way operators justify themselves when accused of being against (or not favoring) the good of the company. This study wants to contribute to highlighting the benefits of the prevention of deviant behavior through a more profound comprehension of its antecedents. The analysis confirms the complexity of human beings, and the need for an integrated approach that leads to clarity and coherence among tools (e.g. mission, code of ethics, incentive system), people and among both people and tools. A central role is played by the dissemination of a culture that considers the firm as a real entity, with its own interest, that is separated from that of its stakeholders and which brings the purpose of the common good.

## Introduction

Financial and environmental scandals have led to a reflection on the motivations that push some individuals to move away from good governance and management, placing them in conflict with the good of the organizations involved and the larger community. This is a cultural crisis that is leading to the deviant behavior of both individuals (Greenberg, 2002) and organizations (Ashforth et al., 2008; Campbell & Göritz, 2014; Levine, 2005; Pinto et al., 2008). This reflection is now of particular importance because of the global economic crisis that has resulted from the *Covid-19* pandemic that follows the global financial crisis of 2008. Many say: “It will never be the same again”.

The way literature and practitioners face the agents’ fraudulent behavior is mainly based on two models of man: rational utility-maximizing agent or irrational and moral agent.

According to the *homo economicus* assumption, the human being is a self-serving individual only interested in maximizing its utility function (Jensen & Meckling, 1976). The choice of keeping an opportunistic or honest behavior is analyzed in terms of costs and benefits: agents act improperly when, rationally, it is more convenient than to behave honestly. They would behave knowingly and deliberately, considering three aspects: 1) the benefit generated by opportunistic behavior; 2) the probability of being discovered, strictly dependent on the control system; 3) the extent of the penalty if discovered (Mazar et al., 2008). Based on these inputs, one would arrive at the choice that maximizes his/her well-being.

This model of human being suggests two remedies to guide the behavior of individuals (Schwartz, 2011): incentives, that push towards an increase of performance, and monitoring of behaviors. Shareholders will benefit from the egoistic managers’ behavior ‘only as by-products of self-interested actions taken under incentive and monitoring regimes … that properly align managerial and shareholder interests’ (Jones et al., 2007, p. 147). Egoism is considered the right thing to have for the well-being of society. Thus, according to this “amoral model”, it seems that the inadequacy of the incentive and monitoring systems is the main motivation of the deviant behavior and it could lead to several justifications when an individual is accused of standing against the corporate interest (Jones et al., 2007). Through the ethical justification he/she demonstrates with valid reasons, to third parties, the regularity and fairness, or even the usefulness and inevitability of his/her action.

However, the human being is not only motivated by selfishness, being also oriented to serve others (Batson, 2014). A pessimistic view of humans discourages virtuous behavior and the reactions can only be consistent with this idea, in the sense that also an altruistic may be transformed into an egoistic person (MacGregor, 1960).

Using the psychological approach, the deviant behavior receives further interpretations, in the sense that it could be the result of intuitive processes that prevent people from recognizing what they are doing (Bazerman & Tenbrunsel, 2011; Sezer et al., 2015). Indeed, human being is much more complex, being both an irrational and moral agent. Blind spots may be present (Bazerman & Tenbrunsel, 2011; Chugh et al., 2005), often due to situational factors and human irrationality, which do not allow the ethical decision-making process to take into account the moral variable (Sezer et al., 2015). Thus, deviant behaviors would not be only the product of rational and conscious choices, or an absolute lack of morality and/or greed of the individuals involved (e.g., Bazerman & Tenbrunsel, 2011).

Although an individual is aware of his/her immoral behavior – therefore situational factors are not present, or, although present they cannot completely obscure his/her morality –, he/she may decide to maintain immoral conduct, rationalizing it (Sykes & Matza, 1957). Moral rationalization allows people to convince themselves that the preference for unethical choice is in line with their moral values ​​(Murphy & Dacin, 2011; Tsang, 2002). According to Tsang (2002), the main assumption of moral rationalization is that people feel the need to perceive their actions to be aligned with their moral standards. Individuals learn the value of moral principles throughout their existence, giving rise to the need to feel they are good people. When something goes against their morality, they experience a negative feeling, the so-called moral cost (Aronson, 1969; Festinger, 1957). In this sense, moral rationalization is motivated by an attempt to remove this sense of guilt.

Consequently, understanding that one must pursue the good of the company and how to pursue it, is still only half of the matter, doing it concretely (and continuing to do it) is the other half. According to Coda: ‘understanding what is good for the company, and doing it, is a guiding principle of common sense, easy to share superficially, but difficult to apply and often in fact disregarded in the life of companies of any type’ (2012, p. 76, our translation). Indeed, ‘the behavior does not automatically follow the decision-making process and in many situations acting ethically can be more difficult than deciding ethically’ (Brady & Logsdon, 1988, pp. 708–709, our translation).

Literature on ethical decision-making processes shows that the orientation of individuals’ behavior towards moral issues is based, among other things, on considerations deriving from a series of ethical theories (Bazerman & Gino, 2012; de Colle & Werhane, 2008; Dion, 2012; Jones et al., 2007). Utilitarianism (of the act and rule), deontology, and virtue ethics are some of them. The same behavior can be considered right by some and wrong by others because of the different ethical approaches.

The paper proposes an integrative model based on a review of literature related to psychological and non-psychological factors, ethical reasoning and ethical justifications. The model recognizes the roles of psychological and non-psychological factors on the link between ethical theories and the ethical reasoning and, consequently, on the way operators justify themselves when accused of being against (or not favoring) the good of the company.

The work is structured in the following way. Section two defines the concept of deviant behavior against (or not in favor of) the good of the company. The psychological and non-psychological factors of deviant behaviors and the possible ethical justifications of violators are analyzed respectively in Sections three and four. The last section is dedicated to the final considerations.

## Deviant Behavior Against (or not in favor of) the Good of the Company

Before discussing the antecedents of deviant behaviors against the good of the company, it is necessary to clarify what this good is. The meaning of the term “good of the firm” derives from the nature of the firm and its objective. According to the shareholder theory, being a legal fiction the company does not exist as a separate institution and its good coincide with the good of its shareholders, considered as owners of the firm (Friedman, 1970), and normally the desire of shareholders is to make as much money as possible.

The stakeholder theory posits that the company should create value not only for shareholders but for all stakeholders of the firm (Freeman, 1984). Then, the good of the company is the good for all its stakeholders.

Undoubtedly, one of the major criticisms that characterize the debate on corporate finalism is precisely the opportunity to recognize an interest to the company (Blair & Stout, 2001; Davies et al., 2004; Mark, 1987; Moore, 2005; Stout, 2012; Velasquez, 1983), which is distinct from the particular (and sometimes temporary) interests of its stakeholders, shareholders included.

Unlike the settings that move between shareholder and stakeholder theory, according to the real entity theory the firm is an entity separated from all its stakeholders (Allen, 1992; Arthur, 1987; Chassagnon & Hollandts, 2014; Gindis, 2009; Lan & Heracleous, 2010; Lozano et al., 2015): ‘[w]hen a company is formed by the union of natural persons, a new real person, a real corporate ‘organism,’ is brought into being… The corporate organism is an animal: it possesses organs like a human being. It is endowed with a will and with senses.’ (Machen, 1911, p. 256). Being a real entity, the firm has its interests and morality, its own rights and duties (Ripken, 2009). According to this approach the firm is not owned by the shareholders and its good is more than just the sum of the goods of its stakeholders (Lozano et al., 2015). Indeed, only a superior good can include simultaneously the economic, social and environmental dimensions of the business. The good of the company must therefore necessarily be multidimensional, as it must simultaneously take into account the interests of stakeholders, company and those of the wider community in which it operates (Melé, 2008). This implies understanding deeply what these interests are and making them compatible.

This aspect is particularly relevant for big corporations, because of the elevated numbers of competing interests and their impact on stakeholders and community. The more intense the company’s growth, the greater should be the necessity of its institutionalization and personification (organization as a person) (Mark, 1987), which tends to have its character as if it were a real person (Davies et al., 2004; Moore, 2005).

In this paper, the good of the company, considered as a real entity, consists in ‘producing useful goods and services, and producing them efficiently (so as to create wealth) and sustainably, so as to guarantee the conditions in which each participant receives from the company what he or she can reasonably expect’ (Argandoña, 1998, p. 1097). Considering the good of the firm as a societal standard, the deviant behaviors concern both those that stakeholders hold against the company for private gains (e.g. an employee steals corporate assets), and those that the company holds against its stakeholders (e.g. insufficient investment in safety in the workplace to maximize profits) and the wider community (e.g. corruption of public officials or pollution). Moreover, the term ‘deviant behavior (or conduct)’ refers to those who do not favor the good of the company (e.g. resistance to change, absenteeism) (Agboola & Salawu, 2011).

According to this logic, the profitability of the firm should not be underestimated. Even if the profit cannot be considered as the purpose of the firm, it is a condition for its survival and growth (especially the undistributed profit), and thus a duty for its community. ‘Consider the profit, according to its true nature, as an indispensable means of the existence and growth of the firm; it also allows the removal of every weapon from those critics (…) who would attack it in moral terms, seeing it as something reprehensible’ (Cassandro, 1969, p. 828, our translation). When the firm survives, due to its profitability, creating value for its stakeholders and community, it can be said that the firm serves the common good (Signori & Rusconi, 2009).

## Psychological and Non-Psychological Factors of Deviant Behaviors

Factors that lead towards deviant behaviors can be classified into psychological (Table 1, A, B and C) and non-psychological (Table 1, D, E and F).

**Table 1:**
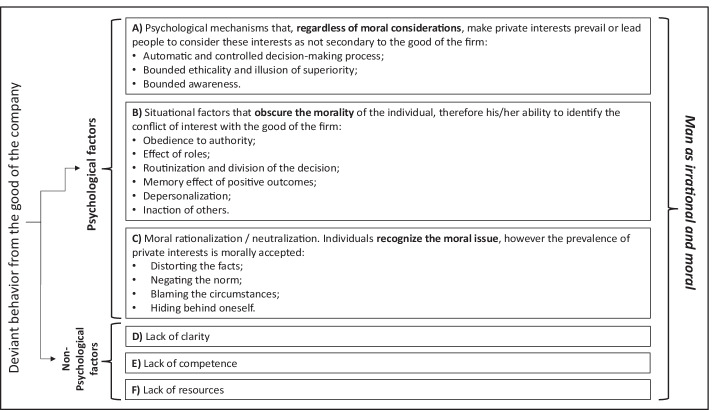
Psychological and non-psychological factors

Source: Author’s elaboration.

### Psychological Factors

There are three categories of psychological factors.

The first (Table 1, A) includes all those that do not consider the individual’s moral aspect, but his/her irrationality. They concern the inability to understand the presence of private interests that interfere with the good of the company, as in the case of conflict of interest, that can be defined as a situation where there is a private interest that tends to interfere with the interest of the firm (Resnik, 1998).

The literature has analyzed some mechanisms that do not allow the individual to perceive that his/her behavior is wrong: automatic and controlled decision-making processes (or dual-mental process, Moore & Loewenstein, 2004; Sezer et al., 2015); bounded ethicality and illusion of superiority (Chugh et al., 2005; Messick & Bazerman, 1996); bounded awareness (Bazerman & Sezer, 2016; Chugh & Bazerman, 2007; Denes-Raj & Epstein, 1994; Gino, 2015; Palazzo et al., 2012).

The automatic processes derive from heuristics (Abatecola, 2014; Gigerenzer & Gaissmaier, 2011), which occur when, in the absence of time and information, the brain tends to simplify the decision-making process. Controlled processes, on the other hand, are the emblem of the cost–benefit analysis that theorists mistakenly assume as the basis for decision-making processes. Controlled processes have a number of characteristics: they are slow, because they are characterized by successive symbol manipulation steps; they are often subjective and require some effort; they are voluntary, in the sense of being deliberately induced and, to a certain extent, they can be deliberately reduced. According to Moore and Loewenstein (2004), when the interests of the company conflict with the agent’s private interests, they are processed differently from the individual: private interests are more subject to automatic processes, while the interest of the firm towards controlled processes. This is one of the reasons why in conflict of interest situations private interests tend to prevail.

Even the incentive system can obscure the ability to morally evaluate one’s actions (Osterloh & Frey, 2004), especially when it is based on extrinsic goods (e.g. bonuses, promotions, Heath, 2009; Kunda, 1990). These incentives activate automatic processes that push towards the private interest in obtaining them, prevailing over the controlled ones.

Chugh et al. (2005) focused their attention on the nature of bounded rationality and its implications in the recognition of the conflict of interest situations, stating that, in some cases, individuals have an excessively positive consideration of themselves. In particular, the authors state that individuals tend to believe they are more moral, competent and worthier than others, or better than the average people. This illusion of superiority limits the ability of people to recognize their conflicts of interest. With regard to competence, the individual tends to perceive him/herself as superior to others, in terms of cooperation, decision-making skills, rationality, intelligence and so on (i.e. self as competent). This leads the individual to judge what is right or wrong defining standards of behavior to favor his/her attributes (Babcock & Loewenstein, 1997).

Bounded awareness (Chugh & Bazerman, 2007, p. 1) does not allow individuals to realize that they are acting against the good of the company, or even against their own interests (Bazerman & Sezer, 2016; Palazzo et al., 2012). This inability can also derive from the fact that a certain phenomenon is normalized, becoming routine (Ashforth & Anand, 2003). The risk is that, although it is clear what the good of the company is, blind spots remain when concrete action is needed to pursue that good (Bazerman & Sezer, 2016). This is the case of the founder who is not aware of the risk in leaving the company governance to an incompetent family member (Chua et al., 2003; Cooper et al., 2013), despite the information on his/her incapacities being evident and available.

In the second and third categories (Table 1, B and C) moral judgment is present in the decision-making process, in the sense that the individual asks him/herself the moral question, which arises when the action of a person can harm or benefit other people (Jones, 1991, p. 367) or the firm in the case it is considered to be a community of persons (Melé, 2012). However, even if he/she is not predisposed to deviant behaviors he/she acts dishonestly (Ariely, 2012; Bersoff, 1999; Cressey, 1973; Mazar et al., 2008; Murphy & Dacin, 2011; Tsang, 2002; Zimbardo, 2007). For this reason, this phenomenon is also called ‘dishonesty of honest people’ (Ariely, 2012), since being moral agents they would know how to distinguish good from evil, having a moral cost when they choose the latter.

The difference between these two categories lies in the fact that while in the first (B) there are situational factors that obscure the individual’s morality, i.e. the ability to distinguish good from evil (Ashforth & Anand, 2003; Tsang, 2002), in the second (C) the individual is aware of the presence of a private interest that tends to interfere with the corporate good, therefore he/she feels a moral cost that competes with the economic gain that can derive from a certain situation, and to behave in a deviant way he/she must activate mechanisms that allow him/her to neutralize this moral cost (Bandura, 1999; Kaptein & Van Helvoort, 2019; Sykes & Matza, 1957). For example, ‘I extracted private benefits in the short run thinking that in the long run the company might compensate the damage’ (e.g. wishful thinking, optimism on the future*,* Babad & Katz, 1991).

In other words, people justify the deviant behavior by recalling some circumstances. For example, a rationalization mechanism could start from the fact that others, especially those who must lead by example (e.g. directors and top managers, Schwartz et al., 2005), stand against the good of the company (e.g. ‘superiors do worse’). The ethical culture asks for congruence, in the sense that ‘managers should apply organizational standards to their own behavior’ (Kaptein, 2011, p. 517). This also explains why honest people may commit immoral acts (Ariely, 2012; Bazerman & Gino, 2012; Cressey, 1973; Mazar et al., 2008; Murphy & Dacin, 2011; Kaptein & Van Helvoort, 2019; Tsang, 2002; Zimbardo, 2007).

### Non-psychological Factors

The deviant behavior may also derive from non-psychological factors, such as lack of clarity and resources of the firm, and lack of competence of individuals (Table 1, D, E and F). These factors can be sources of misinterpretation and misunderstanding, or inability, that do not allow individuals to act in compliance with the good of the company.

Clarity is one of the corporate virtues. It ‘concerns the extent to which the organization makes clear to managers and employees what kind of ethical behavior is expected of them since vagueness and ambiguity are potential antecedents to unethical behavior’ (Kaptein, 2017a, p. 350). Sometimes the individual is not compliant with the rules and procedures, not because he/she has a private interest in breaking them, but because he/she does not know what they are or they are not clear (e.g. inaccurate, incomplete or complex procedures, Park & Jung, 2003) or coherent (e.g. incoherence between objectives and reward system, Kerr, 1975). However, sometimes rules are not complete simply because there are situations characterized by ethical dilemmas where there is not only one right thing to do.

Thus, individuals deviate from the good of the company because they (Table 1, D):

- do not know *what* it consists of, or have a wrong conception of it;
- do not know *how* to pursue it, or have a wrong conception of how to pursue it;
- believe that to do the good of the company means only not to damage it.

Point a) may include situations such as: ‘I thought that the good of the company was to maximize profit for shareholders’ (Blair, 2002); ‘I used transfer pricing to evade taxes in order to maximize the profitability for shareholders’; ‘I bribe a public official to increase the revenues of my company’ (the so-called corrupted organizations, Ashforth et al., 2008; Campbell & Göritz, 2014; Levine, 2005; Pinto et al., 2008; Umphress et al., 2010). These justifications derive from the idea of the firm as a tool for owners to satisfy their interests (Blair, 2002; Bower & Paine, 2017; Morck, 2008; Stout, 2013). The ‘greater good’ for society is absent, and managers serve the interest of shareholders because they believe it is the only right thing to do. In other words, they favor shareholders even when this goes against the well-being of society and their ethical convictions because they believe that current company law requires them to follow courses of action that maximize the shares’ value (Rose, 2007). Therefore, business leaders make decisions that emphasize legal defensibility, rather than ethics or social responsibility.

Di Carlo (2017) undertook a survey of 18 subsidiaries’ board members of an Italian business group, asking them the meaning they give to the term ‘interest of the firm’ contained in the definition of conflict of interest in their code of conduct. Some were shareholder-oriented, others stakeholder-oriented, while some also considered the well-being of society. Thus, since the company did not specify the concept of ‘interest of the firm’, board members interpreted this term according to their experience and values.

Another case is the lack of clarity in the company rules and procedures (point b): ‘I was accused of not having declared my conflict of interest, but reading the guidelines it was not clear what I should have done in the situation in which I was challenged’ (Di Carlo, 2013). The clarity of goals allows individuals to better deal with pressure and temptations (Pearson, 2012) and remove some ethical justifications (Granitz & Loewy, 2007).

In family firms, damage can derive from a lack of clarity about the role of the family members in the firm (Cooper et al., 2013), called role ambiguity (Litzky et al., 2006). Moreover, tolerance of deviant behavior by superiors or colleagues could undermine clarity. As stated by Kaptein, ‘tolerating wrongdoing would suggest that the wrongdoing is “not that wrong” and creates confusion, thus undermining clarity’ (2011, p. 516).

Point c) concerns the inaction of people who, while not acting for personal gains, do not favor the good of the company, for example because they do not signal the improper conduct of others (e.g. inaction of employees to report wrongdoing, Kaptein, 2011) or delay payment to suppliers.

Some could experience a moral cost only when they harm the organization for private benefits. Indeed, the concepts of ‘wrongdoing’, ‘deviant behavior’, ‘fraud’, and ‘corruption’ are often connected to an abuse of power for personal gain (Philp, 2016). However, the delay of payment to a supplier, due to a lack of diligence of the administrative manager, could be perceived by him/her not as a wrongdoing because of the absence of a personal gain, even if the consequence were worse than stealing an asset of the firm (e.g. a tablet).

Sometimes the wrong behavior comes from inappropriate rules (Park & Jung, 2003), such as in a hospital when a wrong protocol is used to identify the symptoms of a virus. Thus, any incompetence for doing the good of the company may also lead to inappropriate rules.

The inability to pursue the company’s good could also result from the incompetence (Table 1, E) of the agent (Rose-Ackerman, 2001), leading to justifications such as: ‘I purchased raw materials paying too much because of my incompetence in dealing with suppliers’ (Ott & Shafritz, 1994). Nepotism and cronyism could be the causes of the presence of incompetent individuals within organizations.

Incompetence can be worse than egoism, because ‘[i]f competency is required to recognize incompetence, truly incompetent people will be both incompetent and unaware of their incompetence’ (Pennycook et al., 2017, p. 1774). Competence is here defined as the ability to achieve the good of the firm.

Thus, deviant behavior could also derive from an honest but incompetent man. ‘The principal may not be fully competent to explain his goals to the agent, and the agent may not be fully competent to understand those goals and to know how to behave in order to maximize the principal’s welfare’ (Kauppi & Van Raaij, 2015, p. 958). Thus, if superiors (directors, top management team) are not able to explain the goals of the company (or its objectives), employees will be confused. The confusion could also derive from the different interpretations people give to the same term. For instance, the term ‘interest of the company’ could be understood in different ways (e.g. interest of shareholder, interest of stakeholder) (Di Carlo, 2017).

Moreover, even if competent, the individual may not have time to serve the good of the company, as in case of busy directors (Fich & Shivdasani, 2012).

The inability to act for the good of the company could then derive from the lack of resources (Table 1, F), even of time (which generates the so-called conflict of commitments, Werhane & Doering, 1995), which does not allow achieving the assigned goals, thus increasing the risk of wrongdoing. As state by Kaptein (2011), achievability is one of the dimensions of the ethical culture of organizations, and it is referred to as the ‘extent to which the organization makes sufficient time, budgets, equipment, information, and authority available to enable employees to fulfill their responsibilities’ (p. 518). For example, ‘I do not have time (or sufficient budget) to achieve all the requested goals’. During the *coronavirus* period, some hospitals did not have enough tools to use in their struggle with the virus (e.g. intensive care beds, swabs, protective masks).

### Interaction Between Psychological and Non-psychological Factors

Psychological and non-psychological factors may affect each other. For instance, the lack of clarity, competences and resources may amplify the inability to understand the presence of private interests (Table 1, A), obscure the morality of individuals (Table 1, C) and increase the moral rationalization (Table 1, B).

For instance, the cost–benefit analysis, activated by the controlled process (Table 1, A), could be limited due to the incompetence of the subject involved and/or lack of clarity of the firm (e.g. of rules and procedures). An incompetent person will obey more easily his/her authority especially if that person has to return a favor to the authority (e.g. in the case of nepotism).

The incompetence may not allow undertaking a cost/benefit analysis for the good of the company, favoring the automatic process in the case of conflicts of interest. Moreover, the lack of competence and time leads to poorer decision-making processes, favoring man’s tendency to simplify (Gigerenzer & Gaissmaier, 2011).

If the firm is not clear that its good does not coincide with the good of shareholders, the tendency of the agent to favor the shareholder could be amplified by some situational factors (Table 1, B) that obscure the morality of the individual (Murphy & Dacin, 2011; Tsang, 2002). In the presence of these factors (e.g. obedience to the authority of the CEO or of the controlling shareholder, Milgram, 1974; Morck, 2008; effect of roles, Zimbardo, 2007), people are unable to understand that their behavior is guided by a particular interest.

Lack of resources (e.g. information), competence and clarity (e.g. of information) could activate some rationalization mechanisms (Table 1, C). For example, an independent director could say that he/she is not able to exercises his/her independence because of a lack of information or clarity, blaming the chairman of the board for that (Brooks et al., 2009).

## Integrative Model of Ethical Justifications of Deviant Behaviors

Ethical theories describe the ethical reasoning of individuals. They have been used to evaluate their implications for corporate finalism (Garriga & Melé, 2004; Jones et al., 2007; Singer, 2010); leadership style (Dion, 2012); preparation of ethics programs (de Colle & Werhane, 2008); arguments that managers use to justify their actions (Lahdesmaki, 2005); students’ attitude towards plagiarism (Granitz & Loewy, 2007); and whistleblowing (Chiu, 2003).

Different ethical reasonings may come to differently define ethical behaviour and consequently deviant behaviour, through a process that involves both psychological and non-psychological factors. The proposed integrative model (see Table 2) posits that psychological and non-psychological factors affect the link between ethical theories and the ethical reasoning and, consequently, the way individuals justify themselves when accused of being against (or not favoring) the good of the company.

**Table 2:**
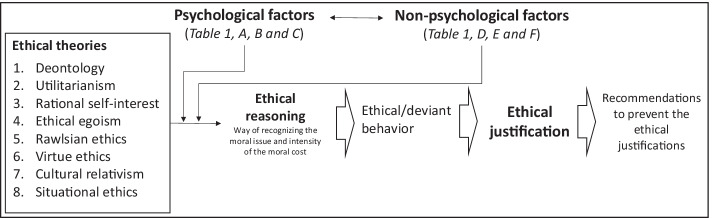
Integrative model of ethical justifications of deviant behaviors

Source: Author’s elaboration.

For each ethical theory one or more ethical justifications can be associated, as well as specific recommendations aimed at preventing these justifications (Granitz & Loewy, 2007). Thus, the same deviant behavior can be attributed to different ways of reasoning and therefore requires different preventive remedies.

It could happen that only after being caught the operator realizes the factors that have determined his/her wrong behavior, for instance because he/she is informed on the reason why it was wrong. According to the proposed model, from the justifications of the operator it can be understood not only the ethical theories that affect his/her ethical reasonings (Granitz & Loewy, 2007) but also the psychological and non-psychological as well as their magnitude.

For instance, according to Granitz and Loewy (2007) students accused of plagiarism, who follow the deontology ethics (‘they can only plagiarize if they misunderstand or are unaware of the theory’, p. 297), may justify their deviant behavior saying: “I didn’t know what plagiarism was”/ “I didn’t know that plagiarism was wrong” (2007, p. 297). From those justifications emerges that the lack of competence of the student and the lack of clarity of the school (Table 1, D and E) affect the ethical reasoning of the former that is based on deontology ethics. Some others justify the plagiarism rationalizing their behavior: “My kid was sick” (Table 1, C).

The analysis of the literature on ethical reasoning has led us to include the possible ethical justifications of those who stand against the good of the company, classified on the basis of eight ethical theories. The selected theories are based on the assumption that human being is not always rational, but also irrational or emotional, as confirmed by the discussed psychological factors that affect behavior.

These justifications can be brought, for example, by the employee to his/her superior, by the chief executive officer to the controlling shareholder, or to the judge due to a trial involving him/her (e.g. for a corruption offense). Each justification can be influenced by psychological and non-psychological factors as well as from their interaction.

Deontology (also called Kantian ethics) bases the moral obligation on the intrinsic value of the action, regardless of the concept of good and the assessment of the extent of the consequences. According to Kant (the so-called categorical imperative): ‘Act only according to that maxim by which you can at the same time will that it should become a universal law’ (1785/1998, p. 422).

Consequently, in deontological reasoning, there are psychological and non-psychological factors that do not allow people to pursue their moral obligation to serve the good of the company. For example: ‘I was incentivized on revenues and this did not make me realize that I was deceiving the customer, going against the principle of honesty of the company’ (Table 1, A); ‘In my university the career advancement is based on publications in A-class journals and this has led me unknowingly to falsify the results of a research to achieve this advancement, failing the institutional interest of the integrity of the research’ (the so-called publish or perish phenomenon in scientific research, Aytug et al., 2019). ‘I did not know that the corruption of a public official for increasing the revenues is against the good of the company’ (Table 1, E, lack of competence).

This leads to the following proposition:

- *Proposition 1: In deontological reasoning, psychological and non-psychological factors may remove the possibility for individuals to be aware of their deviant behavior.*

Utilitarianism falls within the context of consequentialist ethical theories that define the moral correctness of an option by only considering the consequences it generates, i.e., their value or disvalue. Utilitarianism focuses not on action but on its consequences (Arjoon et al., 2018). Utilitarian ethics aim at greater well-being for the highest number of people, therefore precisely at maximizing utility (Bentham, 1789/1996; Hume, 1740/2000; Mill, 1863/1998). It follows that there are no options that are absolutely prohibited or necessary, as the achievement of the best consequences could allow any type of option, even if morally unacceptable, such as that of doing harmful experiments on one person to save many others.

The utilitarian approach can lead to carrying out operations that are against the good of the company because it is mistakenly believed:

- to favor the company without damaging its stakeholders and the community;
- to favor stakeholders and the community without damaging the company;
- that the cost/benefit analysis refers to a specific stakeholder (e.g. the shareholder);
- that the cost/benefit analysis is mainly related to the fraudulent behavior against the company, not also to the responsible behavior for the good of the company.

For point a), the following examples are proposed: ‘I delayed the payment to the supplier because I did not think it would have any effect on its durability’ (inability to identify the consequences from present actions); ‘I did not think that illegal waste disposal could generate certain consequences on the environment’; ‘I made the employees work extremely hard because I didn’t think they would come to a similar stressful situation and, to compensate, to stand against the company’ (Cressey, 1973).

For point b), the authors of the misconduct simply deny (or minimize), even to themselves, the damage (Table 1, C): ‘I took a ream of paper because this has an insignificant effect on the company’ (minimize the consequences). As stated by Horning, ‘they (the employees) wouldn’t come into your home and take thirty cents, but they will take from the Company. They figure it’s got plenty of money and a few cents don’t mean nothing to them’ (1970, p. 55).

Point c) refers to all those cases in which the company is considered as an instrument of the controlling party (e.g. dominant shareholders) to favor its personal interest (Friedman, 1970), reducing the perception of the damage generated to the company in the event of extraction of private benefits. Non-psychological factors, such as the clarity of the firm, or competence of the agent, could be the causes of these justifications.

Finally, point d) refers to those cases in which the person does not realize how much his/her inaction can cause damage to the firm. For example, not paying a supplier on time can generate more damage than taking home a company asset (e.g. a ream of paper). Normally, however, only the latter is considered as behavior against the good of the company.

Utilitarian decision making is a complex cognitive activity (Verbeke et al., 1996) that needs competence. The inability to calculate costs and benefits, therefore the consequences in the short and long term of a given behavior, could depend, among other things, on the: lack of clarity of the firm (Table 1, D), incompetence of the person involved (Table 1, E), and limited information available (Table 1, F). For example, ‘I allowed the controlling shareholder to extract private benefits because I did not know the effect this would have on business continuity’, or ‘I did not evaluate correctly the topic because the chairman of the board of directors did not promptly send me the documentation to be discussed in the board meeting’.

This leads to the following proposition:

- *Proposition 2: In utilitarianism reasoning, psychological and non-psychological factors may not allow the calculation of costs and benefits for the good of the company.*

Rational self-interest (Ashworth & Bannister, 1997) leads one to argue that it is right to take care of his/her private interest at the expense of the corporate interest or take care of the interest of the company against the interest of community, in the presence of psychological factors and non-psychological factors that allow the rationalization of certain opportunistic behaviors or the neutralizing of the moral cost. The following are some possible examples: ‘Owners and managers use the company as their personal ATM, why should I behave correctly?’; ‘My colleagues do worse’ (social comparison processes, advantageous comparison, Festinger, 1957); ‘I subtract cash from the company, but it was just a loan’ (euphemism, Ashforth & Anand, 2003); ‘The company has been delaying the payment of the salary for days, it does not seem wrong to compensate by bringing home just a ream of paper’ (attribution of blame, Bandura, 1999); ‘My boss is incompetent, this demotivates me to such an extent that my interest prevails’ (displacement of responsibility, Bandura, 1999); ‘I have evaded taxes to favor the company, because taxes are used inefficiently by the State’ (denial victims, the deviant behavior is a form of revenge, Bandura, 1999); ‘I falsified the pollutant emissions test (or the budget) because the boss ordered me to do it’ (obedience to authority, Milgram, 1974); ‘I did not receive the information timely, that is why I did not take the right decision’.

This leads to the next proposition:

- *Proposition 3: In rational self-interest reasoning, psychological and non-psychological factors may favor the rationalization of deviant behavior.*

Ethical egoism focuses on maximizing the individual good, being the attitude of the person who cares only for his/her own good. All that promotes the individual good is therefore right, ‘even if the interests of the corporation and its shareholders, for whom managers nominally work, must be sacrificed’ (Jones et al., 2007, p. 144). This good of individuals is linked to the satisfaction of material and immaterial needs (e.g. relational, friendship, recognition).

Ethical egoism (or altruistic egoism) does not exclude that an egoist can think about the well-being of others (Batson & Shaw, 1991), however helping others is an instrumental aim, as the reason derives from the desire to feel good, or to obtain a personal gain (e.g. ‘I collect the cans on the street to put them in a collecting machine and receive money’; ‘I donate my blood because helping others makes me feel a good person’). For the egoist, there are no scruples about going against the corporate good in favor of him/herself. It follows that the company’s good is only pursued if it favors the egoistic person (e.g. incentives linked to profit).

Two forms of selfishness have been studied (Jones et al., 2007, p. 138): psychological and ethical. The first is descriptive and anthropological, in the sense of considering the individual as self-interested, constantly oriented to give precedence to his/her private interests. The second concerns the so-called normative perspective, as it suggests people act exclusively in their exclusive interest. The latter model, therefore, admits that the individual can be altruistic by nature, but obliged to act selfishly because this would favor the general well-being.

The culture of profit maximization for shareholders (Friedman, 1970) is based on the theory of ethical selfishness. The individual is considered to be amoral, therefore he/she cannot be considered morally guilty for his/her opportunistic behavior (Jones et al., 2007, p. 138). The only responsible is the inadequacy of incentives and monitoring system. The following are some examples: ‘I sell junk financial instruments to incompetent customers, bypassing company procedures, to achieve the bonus. If accused of deception, I blame my manager who only incentivized me on sales and did not make me train on the procedure to be followed’.

Even in the case of egoism, non-psychological factors may be relevant, for instance when the violator is incompetent (Table 1, E) in understanding how his/her behavior negatively affects the continuity of the firm and even his/her interest, amplifying the effect of psychological factors (e.g. bounded awareness, Table 1, A). In other words, the incompetent is not always able to perform in his/her best interests. Thus, a true egoistic should be interested in increasing his/her competences in order not to create problems for him/herself. Some companies have been led into bankruptcy because their controlling shareholder did not realize that the damage generated would lead to a point of no return (e.g. Calisto Tanzi in the Parmalat scandal). It is irrational to expropriate a company to the point of damaging oneself. Indeed, the literature on corporate governance evidences that when the major shareholder expropriates his/her company (e.g. using tunneling transactions), damaging minority shareholders and producing financial distress, he/she finds it convenient to prop up the company, guaranteeing its continuity, and when recovered financially it returns to being the subject of tunneling (Peng et al., 2011).

If the company is unclear or it does not give enough resources, the egoist will find more excuses, shifting the responsibility to others. However, for an egoist person, accusing others is not a form of rationalization, since he/she does not feel a moral cost to neutralize.

This leads to the next proposition:

- *Proposition 4: In ethical egoism reasoning, psychological and non-psychological factors may amplify excuses to accuse others.*

As for the deontological ethics, the ethics of justice also seek to overcome the utilitarian idea that the greatest possible well-being must be pursued for the greatest number of people. The ethics of justice is also called Rawlsian ethics, as it is named after its proponent Rawls (2009), the author of a philosophical essay entitled ‘A Theory of Justice’. According to Rawls, the risk inherent in utilitarian theory is that in some cases there is a tendency not to consider the rights of minorities. The risk that principles of justice chosen go to favor their strengths or to discard their weaknesses is amplified by the fact that people tend to view and interpret events through the lens of their personal interests (Babcock et al., 1995), because of some psychological mechanisms (Table 1, A). Thus, the justice should be based on the choices behind a ‘veil of ignorance’, i.e. an imaginary situation in which individuals are ignorant of their circumstances (therefore the potential advantages and disadvantages of the choice), and can more objectively consider the right thing to do.

This is the case of a manager who is asked to evaluate the increase the employees’ salary when the manager’s incentive is linked to the net income for the period (thus, more salary for employees but less bonus for the manager). Using Rawlsian justice, a manager should choose in the presence of a veil of ignorance, imagining, after the choice, that he/she can find him/herself in the situation in which he/she currently is or in that of the employees. This theory can lead in some cases to increasing the salary, even if it would have a negative effect on profitability of the firm. In this case, justice is referred to people, not the company (appeal to higher loyalties, as profitability has to be sacrificed for more important causes, Bandura, 1999), even if the reduction of profit could have negative consequences on the business continuity and growth.

This leads to the next proposition:

- *Proposition 5: In ethics of justice reasoning, psychological and non-psychological factors may confuse the sense of justice for the good of the firm.*

Virtue ethics (Arjoon et al., 2018) is the ethical theory at the basis of the theory of the common good (Argandoña, 1998; Kennedy, 2006; Naughton et al., 1996; Sison, 2007; Sison & Fontrodona, 2011, 2012, 2013; Treviño & Nelson, 2011). Aristotle (1980) is the founding father of that theory.

Virtue ethics considers a certain behavior as good not because it is convenient from the utilitarian point of view or for the respect of a rule, typical of deontology, but because it is the person involved who considers it right as it produces happiness. Virtuousness has roots in the Latin word *virtus*, meaning “strength” or “excellence.” Originally, Plato and Aristotle described virtues as the desires and actions that produce personal and social good.  '"Virtuousness refers to the ideal state of excellence in a human or organizational character, while “virtues” are the specific manifestations of a particular type of character excellence’ (Bright et al., 2006, p. 251).

It follows that while utilitarianism evaluates the consequences, not the actions that determine them, and deontology evaluates the rules, not the consequences that they determine, in virtue ethics the focus is on the ability of actions to produce good for others. The virtuous person acts spontaneously in the right way, by his/her character, without thinking too much about the why of what he/she is doing.

Virtue ethics focuses on the character of people, therefore on their virtues and attitudes. ‘The theory of virtue believes that the foundation of morality lies in the development of good character traits such as virtues: a person is said to be good if he has virtues’ (Arjoon, 2000, p. 161).

The risk for the good of the company is that virtuous people are oriented towards the good of persons without any limit, such as that imposed by company efficiency for its survival and growth (Argandoña, 1998). In other words, being virtuous for the good of people could conflict with being virtuous for the good of the company, which means balancing people’s well-being with business continuity. As stated by Melé ‘management must balance all stakeholder interests and, at the same time, must maintain the survival of the firm’ (2008, p. 10). ‘Humanistic management does not reject efficiency consideration as an organizational goal, but goes beyond it by focusing on people, their dignity and on a continuous process of their development as human beings’ (Roszkowska & Melé, 2020). However, efficiency is only one aspect, since the continuity of the firm asks for a competence that considers simultaneously economic, financial, monetary, and ethical aspects (Di Carlo, 2020).

Possible examples are: ‘I spent too much time looking after a certain stakeholder (e.g. employees) because I am overly empathetic – excess of virtues, or good –’ (Kaptein, 2017b); ‘In cases where I had to choose between man and machine, I have always chosen to safeguard the former, even if this choice has determined the company crisis’ (investment in machines have to be sacrificed for greater loyalty towards employees, Bandura, 1999)’; ‘I made a donation for an important cause, going against the legitimate interests of shareholders to obtain the right dividend’. This latter case could also represent a way for managers to extract private benefits, for instance when they finance the philanthropy initiatives of their family members (Jensen, 2001).

In addition, if the company is intended as a tool to pursue the shareholders’ interest (Friedman, 1970), for the sake of the latter it could lead to accepting behavior against business continuity, especially when oriented towards the short term. According to the stewardship theory (Donaldson & Davis, 1991), there is no potential risk of managerial misbehavior, as managers are eager to be good stewards of shareholders. For example, ‘for the sake of the shareholders I increased the debt (leverage effect), exposing the company excessively to the risk of default’. This behavior can also be fueled by an incentive for managers that is linked to corporate profitability (e.g. stock options, Dong et al., 2010). Thus, the interests of managers can be aligned with those of shareholders, however the interests of the latter may not be in line with the continuity of the firm.

This leads to the next proposition:

- *Proposition 6: In virtue ethics reasoning, psychological and non-psychological factors may confuse the good of people with the good of the firm.*

Cultural relativism (or simply relativism) refers to an inadequate individual culture, matured from past experiences, which pushes towards an unsustainable firm objective. For example, those who have been influenced for years, in universities and business schools, by the theory of profit maximization, that is now heavily condemned (Bower & Paine, 2017; Ghoshal, 2005).

It follows that, culturally, these persons could consider the company as a tool for the owners to maximize the return of their investments. In this logic, the ethical justification is founded in everything that satisfies the shareholders’ desire. If the manager or employee is faced with a choice ‘shareholder vs. stakeholder’ or ‘shareholder vs. collectivity’, the shareholder theory suggests pursuing the shareholders’ interests, since in the long-run this would be the best solution among those available, even in the interests of the community.

Therefore, a manager could say, for example, that in his/her previous companies he/she never had problems favoring shareholders, thereby also positioning him/herself against the interest of other stakeholders (e.g. customers, employees). Lack of clarity from the firm on what is the interest to achieve does not allow compensating for this cultural approach. Moreover, cultural relativism may favorite psychological factors that obscure the morality of the agent. It could be the case of an employee that obeys the authority of his/her superior who required him/her to cheat a customer in order to maximize the profit for shareholders.

This leads to the next proposition:

- *Proposition 7: In cultural relativism reasoning, psychological and non-psychological factors may not allow the removal of the previous culture.*

Situational ethics (Banerjee et al., 1998) regards justifications connected to particular times that the company is experiencing, such as those of a financial crisis (e.g. due to the coronavirus pandemic), which can lead some to not respect the principles of correct management or to have ethical dilemmas that are not easy to solve (e.g. a physician in a private hospital: ‘I recommend unnecessary surgeries or prescriptions to patients because the hospital is in financial distress’, Weiss, 2018). In other words, situations may arise that tempt people to behave wrongly to safeguard the company’s durability or its profitability (e.g. the Volkswagen group emissions scandal, Elson et al., 2015). ‘The company was in crisis, so I decided to deceive customers for the survival of the business’ (Hoffman et al., 1998). Appealing to another norm (Table 1, C), perpetrators can neutralize the violation by appealing to a higher, more important norm that needs to be followed: ‘The customer had been sacrificed for the survival of the firm’ (Kaptein & Van Helvoort, 2019).

In some cases, it is difficult to know what is best for the company because of some paradoxes and contradictions (e.g. ‘Personal versus organizational sustainability agendas’, ‘Corporate short-term versus long-term orientation’, Hahn et al., 2015), and lack of competence and clarity of the firm (or a superior) on how to deal with them.

This leads to the next and final proposition:

- *Proposition 8: In situational ethics reasoning, psychological and non-psychological may confuse the idea of what is right to do.*

## Conclusions

The aim of this paper was to explore what could be the ethical reasoning that operators can use when they are accused of being against (or not favoring) the good of the company, determining which ethical theory characterizes this reasoning, and which psychological and non-psychological factors can have an effect on it. The good of the company has been considered as a good that harmonizes the purposes of individuals, firms, and society.

The study has theoretical and practical implications.

From the theoretical point of view, it contributes to highlighting the benefits of the prevention of deviant behavior through a more profound comprehension of its antecedents, in particular of psychological and non-psychological factors that can influence the ethical reasoning, leading honest people to behave dishonestly (Ariely, 2012; Mazar et al., 2008).

The practical implications are strongly connected to the recommendations to prevent the ethical justifications.

Some ethical justifications show that deviant behaviors do not derive from the intention to extract private benefits, but from the lack of clarity of the firm, irrationality, incompetence or excess of good of the person involved, which does not allow him/her to understand that he/she is acting against the good of the company. Indeed, sometimes the good of some stakeholders (e.g. owners, employees, the community) is pursued without perceiving how such good can affect negatively the company’s good, i.e. its equilibriums and, in the long run, also the stakeholders themselves that actually are intended to be preserved.

Deviant behavior may derive from the fact that people do not recognize the interest (or good) of the firm as separate from that of its stakeholders, in particular of those who have the power to dominate it (e.g. major shareholder, management), and that this interest should be compatible with the interest of stakeholders as well as with that of the wider society. This inability to recognize the interest of the firm may come from a culture in line with shareholder and stakeholder theories, according to which the firm is a legal fiction without its own interests and responsibilities (Lozano et al., 2015).

Thus, the most relevant recommendation seems to be the separation of the interest of the company from that of its stakeholders and to include in this interest the multi-dimensional logic of the common good (Bower & Paine, 2017; Coda, 2012; Di Carlo, 2020; Melé, 2008). Indeed, to prevent some ethical justifications, this separate entity must be considered as a community of persons (Melé, 2012) that does not have only duties towards stakeholders, but also towards the larger collectivity (Argandoña, 1998). Moreover, the real entity has not only duties but also rights. Stakeholders should not only use the company as the instrument for their good, having also duties in serving the good of the company itself.

It is therefore central to clarify: what the good of that real entity is; how to achieve it; how to mitigate or remove the obstacles that make difficult to concretely do it, due to some psychological and non-psychological factors; and what could be the effect of deviant behavior on that good. Honest people may behave dishonestly because they do not have a clear ‘compass’ or, if it is present, they do not know how to use it.

The interest of the corporate community does not eliminate the interests of its members; instead, it asks to make them compatible with the interest of that community, as well as the interest of the latter being compatible with that of the larger collectivity. Virtue ethics is the ethical theory of the common good (Arjoon et al., 2018). According to this ethical approach, the individual is not a means, but an end. The aim of the work is to create conditions that promote his/her well-being (Arjoon, 2000), i.e. the cultural, moral and economic flourishing. This approach is useful to prevent several ethical justifications connected to rational self-interest because it favors the dialogue with stakeholders in order to understand their interests (not only economic) and how to satisfy them.

However, scholars who suggest adopting virtue ethics (Hackett & Wang, 2012) as a leadership style should refer not only to the good (or happiness) of persons but also to that of the company (therefore to a community with a greater interest than that of its members), which must balance, in a dynamic and evolutionary way, the economic, social and environmental aspects. The happiness of persons is closely interrelated to the survival and development of the community to which they belong.

It is not sufficient to underline the importance of doing the good of the firm and to specify what that good is, being also necessary to have specific knowledge to achieve this good. Moving from the paradigm of shareholder value creation to that of the multi-dimensional purpose of the common good, could generate confusion that arises when there is inadequate competence to manage the paradoxes that come from the necessity to achieve simultaneously the economic, social and environmental aspects (Hahn et al., 2015), for instance when in the short-term achieving one aspect (the environmental) could be detrimental for other aspects (e.g. short-term profitability). This confusion could also increase the risk of managerial opportunistic behavior (Jensen, 2001).

To understand the interconnection of the consequences of individuals’ behavior, it is suggested adopting a systemic approach that better allows understanding the complexity of the firm, and the interdependence between its parts and the whole, counterbalancing the tendency of human beings to simplify their reasoning (e.g. due to the heuristics used, Gigerenzer & Gaissmaier, 2011).

Moreover, the logic of the real entity oriented towards the common good asks for a model of man that is different from the logic of the self-interested ‘individual’. The human being is a ‘person’ (not an individual) (Argandoña, 1998, p. 1094) capable of cooperating with a spirit of service, altruism, reciprocity and gratuity. He/she has intrinsic and transcendent motivations to develop, for the common good of the community (hence for him/herself), collaborative relationships.

Many ethical justifications find origin from the egoistic model of man theorized by the agency theory, and from mechanisms normally used to discipline his behavior, such as incentives and monitoring system. When setting goals, leaders must put themselves in the shoes of those whose behavior they try to influence and reflect on their potential reactions (Bazerman & Tenbrunsel, 2011), that generate consequences for the interests of the company. Ethics training and codes of ethics are useless if incentives and leaders push towards achieving a result without wondering how it is achieved. Indeed, even if companies want to promote ethics, they favor anti-ethical behaviors (Bazerman & Tenbrunsel, 2011).

The role modeling of leaders is central and it must consist not only of clearly transmitting to collaborators that they must not harm the company, but also, and above all, that they must strive to do their best for its good.

The company should use an integrated approach/thinking that leads to consistency between tools/rules, between people and both between tools/rules and people. The inconsistency and unclearness of tools and people lead to a weakening of systemic links, questioning the continuity of the company.

### Further Research

The link between ethical justifications and psychological and non-psychological factors must be tested, and in particular how psychological and non-psychological factors may affect each other. Moreover, future research could analyze what are the ethical justifications most used in the various types of organizations (e.g. companies compared to public administrations, family-owned companies compared to State-controlled companies), to identify specific psychological and non-psychological factors that represent the antecedents of deviant behavior, as well as specific recommendations and related tools. Indeed, even though the paper focuses on business entities, results could be easily generalized to other organizations, such as public administrations and non-profit organizations. Moreover, it could be interesting to evaluate if and how individual factors (e.g. sex, age, nationality) affect ethical justifications.

The article focuses on the psychological approach to describe the irrationality of human being. However, being that only one of the approaches, further studies may investigate the effect of other approaches on ethical justifications.

Finally, it could also be interesting to study the effect that the idea of the firm as a community and its orientation on the common good may have on these factors as well as on ethical justifications.
